# An Unusual Case of Cryoglobulinemic Vasculitis and Acute Disseminating Encephalomyelitis in a 21-Year-Old Caucasian Male

**DOI:** 10.7759/cureus.59438

**Published:** 2024-05-01

**Authors:** Saumya L Karne, Pavan Kumar R Panchavati, Naveen K Tangutur, Jyothirmayee Pabbaraju

**Affiliations:** 1 Internal Medicine, Wayne State University, Detroit Medical Center, Detroit, USA; 2 Family Medicine, Huntsville Hospital, Huntsville, USA; 3 Medicine, Huntsville Hospital, Huntsville, USA; 4 Anatomy, SVS Medical College, Mahabubnagar, IND

**Keywords:** intravenous drug abuse, grand mal seizures, cryoglobulinemia vasculitis, staphylococcus lugdunensis, infective endocarditis, acute disseminating encephalomyelitis

## Abstract

Cryoglobulinemic vasculitis and acute disseminated encephalomyelitis (ADEM) are characterized by damage to either blood vessels or grey matter. For both diseases, infections can be an etiology. In cryoglobulinemic vasculitis, the initial insult causes damage to the glomerulus, and in the case of ADEM, damage leads to a central nervous system demyelinating disorder. Infective endocarditis can be associated with both diseases and can be challenging to diagnose. Individuals on antibiotics may present with negative blood cultures, making underlying infective endocarditis difficult to diagnose. In this report, we describe a 21-year-old male who presented to the hospital after an assault with splenic laceration and was subsequently found to have infective endocarditis associated with cryoglobulinemic vasculitis and ADEM.

## Introduction

Cryoglobulinemic vasculitis is a small-vessel disease characterized by damage to the blood vessels from immune complex deposition [1]. Cryoglobulins are present in the blood due to insufficient clearance of immune complexes and can lead to systemic vasculitis [2]. Cryoglobulinemia vasculitis is most often associated with hepatitis C infections, with symptoms ranging from purpura and arthralgia to glomerulonephritis and widespread vasculitis [1,3]. Acute disseminated encephalomyelitis (ADEM) is an inflammatory demyelinating disorder that affects the central nervous system [4]. Etiologies of ADEM may include vaccinations due to infiltration of inflammatory cells that lead to demyelination [5]. Infective endocarditis is associated with IV drug abuse and the incidence of infective-endocarditis-related hospitalizations has increased in the last decade [6,7]. This case presents a patient who presented to the hospital due to an assault but turned out to have both cryoglobulinemia vasculitis and ADEM due to underlying infective endocarditis.

## Case presentation

A 21-year-old Caucasian male, with a past medical history of hemophilia, hepatitis C, and intravenous (IV) drug abuse, presented with a chief complaint of assault with a splenic laceration. The patient was initially started on piperacillin/tazobactam and linezolid and subsequently underwent a splenectomy without any initial complications.

The patient was found to have a non-oliguric acute kidney. Initial urinalysis showed significant glucosuria and large proteinuria with leukocytes present (however, no nitrites or leukocyte esterase), and a kidney biopsy revealed that he had crescentic glomerulonephritis and cryoglobulinemia. Initial diagnosis of hepatitis C-induced cryoglobulinemia was suspected. However, labs revealed a negligible viral load for hepatitis C, arguing against presumed hepatitis C-induced cryoglobulinemia.

**Figure 1:**
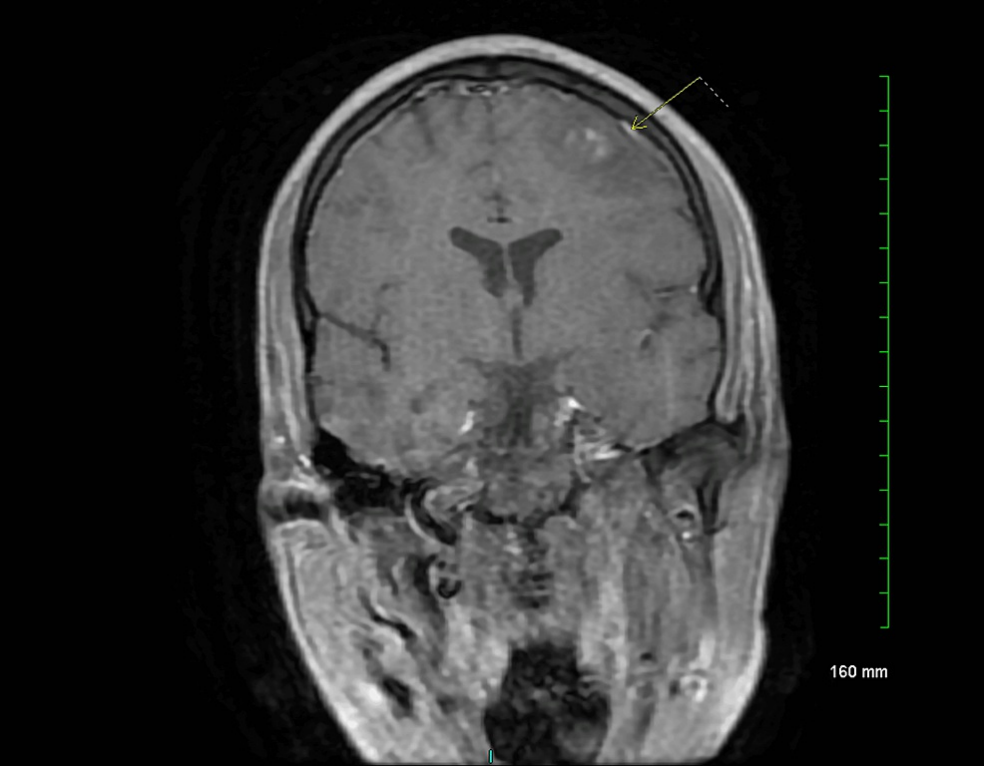
MRI of the brain showing acute disseminated encephalomyelitis (see arrow to identify the lesions)

The patient began experiencing seizures that persisted despite changes in the antiepileptic drug regimen. Further workup ruled out drugs of abuse as the cause of his seizures. As the patient was experiencing recurrent seizures, he was intubated for airway protection. Neurological evaluation revealed acute disseminating encephalomyelitis on magnetic resonance imaging (MRI) (Figure 1). The patient was started on high-dose steroids, and, subsequently, plasmapheresis was initiated.

**Figure 2:**
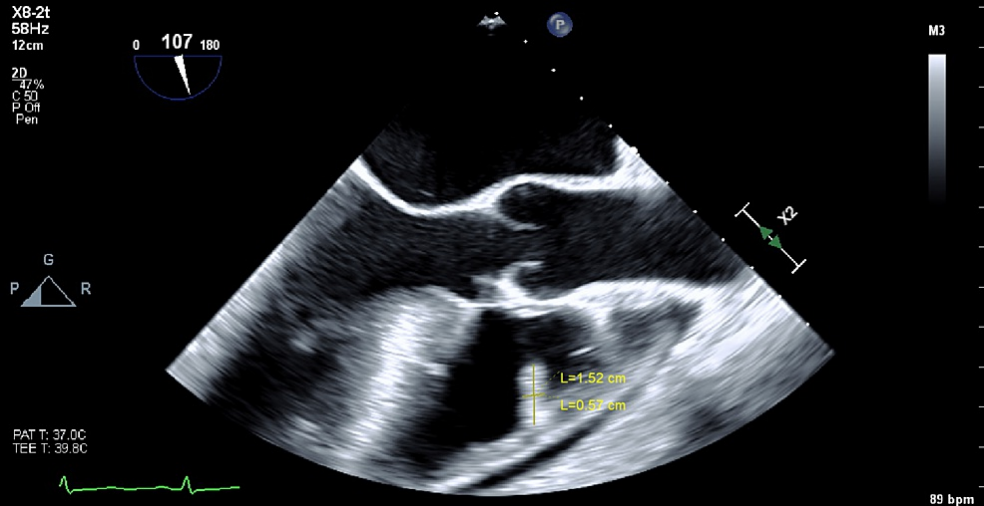
Transesophageal echocardiography (TEE) image showing vegetation (1.52 cm x 0.57 cm) suggestive of a valvular disease

During his stay, the patient developed a fever with leukocytosis. Further evaluation including repeat blood cultures revealed that he had an infection with *Staphylococcus lugdunensis, *and the patient's antibiotic regimen was switched to daptomycin*. *The patient also had an echocardiography showing vegetation, and this was confirmed by the transesophageal echocardiogram (TEE), suggesting a valvular disease (Figure 2). The patient was diagnosed with infective endocarditis. The patient’s renal function improved, seizures were controlled, and his clinical condition improved significantly. He was discharged home on intravenous dalbavancin with home infusion therapy once a week.

The final diagnosis was that the patient's cryoglobulinemia and ADEM were triggered by his subacute infective endocarditis.

## Discussion

Cryoglobulinemia vasculitis is a small-vessel vasculitis that presents with purpura, weakness, myalgia, and arthralgia [3]. More commonly, cryoglobulinemia is associated with hepatitis C infection. However, *Staphylococcus aureus* has also been associated [1]. The most common manifestation is cutaneous purpura, consisting of small petechial lesions in the legs. Renal involvement can present as proteinuria, microscopic hematuria, red-blood cell casts, and renal failure [3]. Previous case reports have described infective endocarditis as the cause of a patient’s cryoglobulinemic vasculitis. In this case, the patient’s symptoms suggestive of an infection were masked, and upon receiving steroids, his clinical condition worsened. Upon discontinuation of steroids and starting antibiotics, the patient’s clinical disposition improved [8,9].

This patient’s stay was also complicated by intractable seizures requiring intubation. It was found that this was due to ADEM, an inflammatory demyelinating disorder affecting the central nervous system [4]. Previous cases have described an infectious etiology to ADEM in which a patient being treated for bacteremia subsequently developed seizures that required intubation [10]. The bacteremia elicits an inflammatory reaction in the blood vessels, causing edema and perivenular demyelination, leading to ADEM [11]. In our patient, underlying untreated infective endocarditis was the cause of ADEM. The patient’s history of IV drug abuse also contributed to the development of ADEM as another case has described such an association [12].

Many of the patient’s symptoms could be attributed to infective endocarditis with *S. lugdunensis*, a coagulase-negative bacteria. This could have been presented when the antibiotics were administered after the assault and splenic removal. Previous studies have described that antibiotic use can sterilize the blood [13]. Though this patient did receive broad-spectrum antibiotics after removal of the spleen that could explain the sterilization of the blood, he did not receive enough antibiotics to treat the underlying infection. A previous study showed that antibiotics need to be administered in high doses for at least four weeks for them to be effective at treating slow-growing bacteria [14,15].

The patient, in this case, had both cryoglobulinemia vasculitis and ADEM associated with underlying infectious endocarditis with *S. lugdunensis*. This case was unique in that the patient presented to the emergency room for a splenic laceration following an assault. He underwent surgery for the removal of the spleen, and afterward, broad-spectrum antibiotics were administered. It is thought that the prophylactically administered broad-spectrum antibiotics served as an inciting factor that sterilized the blood cultures, allowing the bacteria *S. lugdunensis* to grow slowly. In this patient, he presented with both cryoglobulinemia vasculitis and ADEM.

## Conclusions

In conclusion, we present a rare case of infective endocarditis from *S. lugdunensis* as the etiology for cryoglobulinemia vasculitis and ADEM. This presentation was unique in that the patient presented to the hospital with a splenic laceration with negative blood cultures and was diagnosed with acute kidney injury secondary to cryoglobulinemia vasculitis, which was initially thought to be from hepatitis C. He was later diagnosed with subacute infective endocarditis from *S. lugdunensis*, which caused his ADEM and cryoglobulinemia vasculitis.

## References

[1] Lidar M, Lipschitz N, Langevitz P, Shoenfeld Y (2009). The infectious etiology of vasculitis. Autoimmunity.

[2] Ferri C, Zignego AL, Pileri SA (2002). Cryoglobulins. J Clin Pathol.

[3] Ramos-Casals M, Stone JH, Cid MC, Bosch X (2012). The cryoglobulinaemias. Lancet.

[4] Mahdi N, Abdelmalik PA, Curtis M, Bar B (2015). A case of acute disseminated encephalomyelitis in a middle-aged adult. Case Rep Neurol Med.

[5] Filippi M, Rocca MA (2020). Acute disseminated encephalomyelitis. White Matter Diseases.

[6] Cuervo G, Escrihuela-Vidal F, Gudiol C, Carratalà J (2021). Current challenges in the management of infective endocarditis. Front Med (Lausanne).

[7] Alkhouli M, Alqahtani F, Alhajji M, Berzingi CO, Sohail MR (2020). Clinical and economic burden of hospitalizations for infective endocarditis in the United States. Mayo Clin Proc.

[8] Agarwal A, Clements J, Sedmak DD, Imler D, Nahman NS Jr, Orsinelli DA, Hebert LA (1997). Subacute bacterial endocarditis masquerading as type III essential mixed cryoglobulinemia. J Am Soc Nephrol.

[9] Liu K, Newman A (2020). Infective endocarditis observed with cryoglobulinemic vasculitis. Cureus.

[10] Shujaat SD, Durica S (2019). Acute disseminated encephalomyelitis in an adult associated with staphylococcus aureus bacteremia and infective endocarditis (P1.2-038). Neurology.

[11] Alper G (2012). Acute disseminated encephalomyelitis. J Child Neurol.

[12] Hatharasinghe A, Akhondi H, Pepito D (2020). Acute disseminated encephalomyelitis. HCA Healthc J Med.

[13] Hubers SA, DeSimone DC, Gersh BJ, Anavekar NS (2020). Infective endocarditis: a contemporary review. Mayo Clin Proc.

[14] Luque Paz D, Lakbar I, Tattevin P (2021). A review of current treatment strategies for infective endocarditis. Expert Rev Anti Infect Ther.

[15] La Civita L, Fadda P, Olivieri I, Ferri C (2002). Cryoglobulinaemic vasculitis as presenting manifestation of infective endocarditis. Ann Rheum Dis.

